# Chronic Lymphocytic Leukemia with Dermatomyositis: A Therapeutic Challenge

**DOI:** 10.4274/tjh.galenos.2020.2020.0331

**Published:** 2020-11-19

**Authors:** Sudhir Kumar, Ajay Gogia, Ritu Gupta, Soumya Mallick

**Affiliations:** 1 AIIMS, Department of Medical Oncology, New Delhi, India; 2 AIIMS, Department of Laboratory Oncology, New Delhi, India; 3 AIIMS, Department of Pathology, New Delhi, India

**Keywords:** CLL, Cutaneous involvement, Rash

## To the Editor,

Chronic lymphocytic leukemia (CLL) is an indolent B-cell malignancy that is often complicated by autoimmune abnormalities like autoimmune hemolytic anemia and immune thrombocytopenia. Non-hematological autoimmunity occurs in 1%-2% of patients with CLL [1]. Dermatomyositis (DM) is an immune-mediated, inflammatory muscle disease that is reported to be associated with CLL and causes significant clinical dilemmas owing to its rarity. We report a case of CLL diagnosed concurrently with DM, treated with chemoimmunotherapy.

**Figure 1:**
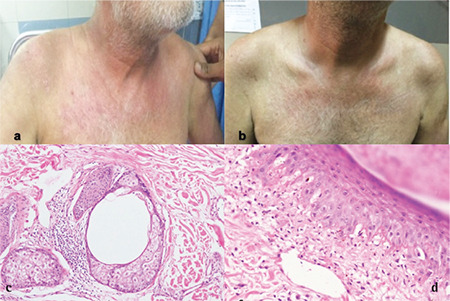
a) Erythematous, irregular, and crusting plaque-like skin rash (shawl sign) before treatment. b) Resolved skin rash after therapy. c, d) Skin biopsy showing focal vacuolar interphase colloid bodies, papillary dermal edema, and dilated capillaries in the upper dermis with elastotic degeneration.

A 60-year-old man presented with a progressive skin rash over the base of the neck, knuckles of both hands, and trunk along with symptoms suggestive of proximal muscle weakness predominantly involving the lower limbs for 4 months. He had been bedridden for the preceding 2 months. On examination, he had erythematous, irregularly shaped plaques with crusting over the neck, upper chest (shawl sign) (Figures 1a and 1b), bilateral knuckles, and periumbilical area without any mucosal lesions. Central nervous system (CNS) examination showed bilaterally symmetrical proximal muscle weakness (grade 3/5), predominantly involving the lower limbs. The rest of the CNS and other systemic examinations were unremarkable. He had hemoglobin of 11 g/dL and leukocytosis with an absolute lymphocyte count of 13224/mm^3^. Peripheral blood smear examination and flow cytometry were compatible with the diagnosis of CLL. The fluorescent in situ hybridization panel for CLL showed 13q deletion. His creatinine kinase level was elevated at 2092 U/L (reference range: 10-225 U/L), as was serum lactate dehydrogenase (557 U/L; reference range: 200-420 U/L). Autoimmune panel, nerve conduction, and electromyographic studies were negative. Skin biopsy showed focal vacuolar interphase colloid bodies, papillary dermal edema, and dilated capillaries in the upper dermis with elastotic degeneration suggestive of DM (Figures 1c and 1d). The diagnosis of CLL (clinical Rai stage 0) with DM was made. He was started on prednisolone at 1 mg/kg and methotrexate at 15 mg/weekly by a dermatologist; however, the rashes were gradually progressive. Due to his uncontrolled rashes after 2 months of steroid and methotrexate treatment and marked recent-onset fatigue, he was put on CLL therapy with a BR regimen (bendamustine at 90 mg/m^2^ and rituximab at 375 mg/m^2 ^q 28 days). He then recovered from the rashes, weakness, and fatigue. He attained complete hematological and clinical remission after six cycles of BR and remains in follow-up and disease-free for the last 18 months.

Among 15 reported cases of CLL with inflammatory myositis in the last two decades, only six were associated with DM [2]. It is not known whether this association is coincidental or causal or merely due to patient selection bias. DM is usually diagnosed concurrently with (5 out of 6 reported cases) or after (1 out of 6) a diagnosis of CLL and it follows a parallel course, suggesting a pathogenic link between the two entities [2]. Tumor antigens triggering the autoimmune process either by mimicking endothelial antigens or via bystander stimulation and imbalance of T-cell subsets in CLL are the most reported hypotheses [3,4]. In general, the treatment of autoimmune complications in CLL has been the same as when the disease occurs spontaneously, but the question of whether and how to treat CLL itself persists. No treatment plan for 5 out of 6 cases of CLL with DM were reported, and one patient received methotrexate with cyclophosphamide. The skin rash in DM does not mean malignant skin involvement; therefore, DM treatment should be initiated separately, as in our case. However, DM with associated malignancy responds poorly compared to idiopathic cases [5]. Long-term outcomes of CLL with DM are unknown, and so are data regarding the use of chemoimmunotherapy in such patients, although rituximab has shown efficacy in DM [6]. It is prudent for the overworked clinician to not overlook neurological or musculoskeletal symptoms in CLL patients, and a keen search for hematological malignancy is justified in known cases of inflammatory myopathies.
